# Evidence of Uncoupling between Renal Dysfunction and Injury in Cardiorenal Syndrome: Insights from the BIONICS Study

**DOI:** 10.1371/journal.pone.0112313

**Published:** 2014-11-11

**Authors:** Matthieu Legrand, Benedetta De Berardinis, Hanna K. Gaggin, Laura Magrini, Arianna Belcher, Benedetta Zancla, Alexandra Femia, Mandy Simon, Shweta Motiwala, Rasika Sambhare, Salvatore Di Somma, Alexandre Mebazaa, Vishal S. Vaidya, James L. Januzzi, from the Global Research on Acute Conditions Team (GREAT)

**Affiliations:** 1 AP-HP, Groupe hospitalier St-Louis-Lariboisière, Department of Anesthesiology and Critical Care and Burn unit, F-75475, Paris, France; 2 Univ Paris Diderot, Paris, France; 3 U942 Inserm F-75475, Paris, France; 4 Emergency Medicine, Department of Medical-Surgery Sciences and Translational Medicine, University Sapienza Rome, Sant’Andrea Hospital, Roma, Italy; 5 Division of Cardiology, Massachusetts General Hospital, Harvard Medical School, Boston, MA, United States of America; 6 Harvard Medical School and Harvard School of Public Health, Boston, MA, United States of America; I2MC INSERM UMR U1048, France

## Abstract

**Objective:**

The objective of the study was to assess urinary biomarkers of renal injury for their individual or collective ability to predict Worsening renal function (WRF) in patients with acutely decompensated heart failure (ADHF).

**Methods:**

In a prospective, blinded international study, 87 emergency department (ED) patients with ADHF were evaluated with biomarkers of cardiac stretch (B type natriuretic peptide [BNP] and its amino terminal equivalent [NT-proBNP], ST2), biomarkers of renal function (creatinine, estimated glomerular filtration rate [eGFR]) and biomarkers of renal injury (plasma neutrophil gelatinase associated lipocalin [pNGAL], urine kidney injury molecule-1 [KIM-1], urine N-acetyl-beta-D-glucosaminidase [NAG], urine Cystatin C, urine fibrinogen). The primary endpoint was WRF.

**Results:**

26% developed WRF; baseline characteristics of subjects who developed WRF were generally comparable to those who did not. Biomarkers of renal function and urine biomarkers of renal injury were not correlated, while urine biomarkers of renal injury correlated between each other. Biomarker concentrations were similar between patients with and without WRF except for baseline BNP. Although plasma NGAL was associated with the combined endpoint, none of the biomarker showed predictive accuracy for WRF.

**Conclusions:**

In ED patients with ADHF, urine biomarkers of renal injury did not predict WRF. Our data suggest that a weak association exists between renal dysfunction and renal injury in this setting (Clinicaltrials.gov NCT#0150153).

## Introduction

Numerous clinical studies have shown a strong association between worsening of renal function (WRF) and mortality in patients with acutely decompensated heart failure (ADHF)[1]. WRF due to ADHF has been described as cardio-renal syndrome (CRS), underlying the interaction between cardiac dysfunction and the risk of renal dysfunction. The role of venous congestion has received much attention in this setting, which makes therapeutic interventions to remove fluid suitable to prevent of treat CRS. Identification of patients with a high risk of WRF therefore appears warranted in order to select patients who could best benefit from specific intervention or tailored therapies. Although baseline renal function and several clinical factors have been associated with WRF, they remain poorly predictive of WRF. In this line, identification of clinical and biological markers that improve diagnostic accuracy and prediction of WRF are needed [2].

WRF is typically recognized after it has occurred, when conventional measures of renal function, such as serum creatinine become abnormal^3^. With the development of biomarkers more specific to renal injury, it may be possible to detect WRF earlier than such standard means, and thus predict WRF before it occurs. Although several studies have shown prognostic importance of several novel biomarkers of renal injury, most have been in patients with chronic HF [2], [4]–[6]. Few have examined the outcome measure of WRF [7], [8], and even fewer have compared multiple candidate biomarkers [9]. While the majority of data in this area has focused on blood-based biomarkers of renal injury, such as neutrophil gelatinase associated lipocalin (NGAL), the presence of urine biomarkers (suggested to more specifically represent renal injury) has not been systematically studied in a comparative manner.

We conducted a prospective multicenter study, with the aim to assess the ability of emerging blood and urinary-based biomarkers of renal injury and function to predict WRF and outcome in ADHF patients.

## Methods

### Study design and setting

The Biomonitoring and Cardiorenal Syndrome in Heart Failure Trial (Clinicaltrials.gov NCT#01570153) enrolled subjects between April and July 2012 at two tertiary care academic medical center members of the Global Research on Acute Conditions Team: the Massachusetts General Hospital (Boston, MA), and Sant’Andrea Hospital (Rome, Italy). The study was approved by the Institutional Review Boards at Partners Healthcare and Sant’Andrea. All patients gave written consent prior to study procedures using institutionally-approved consent forms.

### Selection of Participants

Sequential patients were screened during working hours. Patients with decompensation of chronic HF as well as new-onset HF were included. Inclusion criteria included patients with NYHA Class I–IV symptoms of symptomatic ADHF requiring intensification of diuretic therapy. Exclusion criteria included renal failure requiring current renal replacement therapy, ≥8 hours from first dose of intravenous diuretic, and unwillingness or inability to participate in study procedures.

### Methods and Measurements

Baseline demographics, vital signs, and results of physical examination were recorded after informed consent was signed. Blood was drawn and urine sample collected and processed as noted below.

#### Blood and urine analysis

Blood was drawn into tubes containing ethylenediaminetetraacetic acid or no anticoagulant, and spun for 15 minutes; samples were immediately aliquotted to freezer tubes and frozen at −80° for biomarker measurement following the completion of the trial. In addition, a 10 mL sample of urine was collected. If necessary, a urine sample was obtained from the Foley catheter tube. The urine sample was for 10 minutes, and aliquoted into freezer tubes for biomarkers measurement following the completion of the trial.

Biomarkers of myocardial stretch included amino-terminal pro-B type natriuretic peptide (NT-proBNP; Roche Diagnostics, Indianapolis, IN), BNP (Alere Triage BNP, San Diego CA), and soluble ST2 (Presage ST2, Critical Diagnostics, San Diego, CA).

Biomarkers of renal function included blood urea nitrogen (BUN), serum creatinine (Screat), and estimated glomerular filtration rate (eGFR; estimated using the simplified Modification of Diet in Renal Disease equation). Biomarkers of renal injury included plasma NGAL (Alere, San Diego, CA), urinary Cystatin C (uCyst; Millipore, Billieria, MA), urine fibrinogen (Millipore, Billieria, MA) and urine N-acetyl-beta-D-glucosaminidase (NAG; Roche Diagnostics, Basel, Switzerland). In addition, another marker of renal proximal tubular injury, Kidney Injury Molecule-1 (KIM-1), was measured in the urine using previously established Luminex-based assay and the levels of all urinary biomarkers were normalized to urinary creatinine. Urinary creatinine concentrations were measure using commercially available kit from Cayman Chemical (Ann Arbor, MI) Physicians were blinded of the results of the biomarkers excepted for NT-proBNP and BNP, which were typically drawn for the purposes of standard of care evaluation of HF.

### Outcomes

The primary endpoint of the study was the ability of objective measures to predict WRF defined as a rise of serum creatinine by an absolute change of 0.3 mg/dL or a relative rise ≥25% from baseline within 72 hours from admission. In patients meeting the criteria of WRF, the etiology of renal dysfunction was judged by two study physicians blinded to the results of novel renal biomarkers using standard criteria. Patients with WRF were subsequently characterized using RIFLE classification.

### Analysis

Baseline variables of study participants as a function of WRF were compared using the students T test or *Χ^2^* test as appropriate; the Mann-Whitney U test was used for continuous variables in the states of non-normality. Continuous variables were summarized as mean ± standard deviation if normally distributed, while non-normally distributed continuous variables were summarized as median and inter-quartile range.

Using WRF as the gold standard diagnosis, median biomarker concentrations were compared. Results from biomarker testing were examined as a function of RIFLE classification using the Kruskal-Wallis test. Receiver operating characteristic (ROC) tests compared the results of each biomarker for predicting WRF, expressed as area under the ROC. From ROC testing, an optimal threshold for predicting WRF was identified for candidates.

Univariable comparisons between baseline characteristics were used to identify candidate variables for entry to a multivariable logistic regression model; in both uni- and multivariable models, we used WRF as the dependent variable first, followed by separate analyses for WRF or in-hospital death; only those with a P value <.05 were retained for multivariable modeling. Odds ratios (OR) and 95% confidence intervals (CI) were generated.

In order to better examine the prognostic importance of biomarker combinations, subjects were grouped relative to optimal cut-offs for natriuretic peptides *plus* NGAL, and again examined in uni- and multivariable logistic regression. Rates of WRF as a function of results of candidate predictors were examined.

ROC analyses were performed using Analyse It software (Leeds, UK), while all other statistical analyses were performed using either PASW Statistics Version 17.0 (Chicago, IL, USA) or SAS (Version 9.2; Cary, NC, USA). All P values are two-sided with a value of <0.05 considered significant.

## Results

### Characteristics of study subjects

87 consecutive emergency department patients with ADHF (mean age 74.6±11.5 years) were enrolled with both blood and urine sample. Of those patients enrolled, 53 (56%) had decompensation of prior chronic HF. Of these, 23 (26%) developed WRF. Eight patients died (9.2%) during hospitalization. Of these, 6 (75%) developed WRF prior to dying. The median change in renal function among those dying was +39% (interquartile range 1.0%–82%). Baseline characteristics as a function of WRF are detailed in **Table 1**. Demographic characteristics and clinical presentation did not differ between patients with and without WRF, except for higher frequency of pleural effusion on chest radiography among those who later developed WRF.

**Table 1:** Baseline characteristics of study subjects as a function of the subsequent development of WRF.

Characteristic	WRF (N = 23)	No WRF (N = 64)	P
Age, years, mean ± SD	74.6 (10.1)	74.6 (11.7)	0.99
Gender, male, n (%)	16 (69.6%)	9 (60.9%)	0.46
Caucasian, n (%)	23 (100.0%)	57 (89.1%)	0.10
**ED Presentation**			
Chest pain, n (%)	16 (25.0%)	4 (17.4%)	0.46
Paroxysmal nocturnal dyspnea, n (%)	4 (17.4%)	19 (29.7%)	0.25
Orthopnea, n (%)	18 (78.3%)	41 (64.1%)	0.21
**Medical History**			
LVEF, %, mean ± SD	48.0 (14.7)	55.3 (17.1)	0.15
Chronic kidney disease, n (%)	9 (39.1%)	20 (31.3%)	0.49
Hypertension, n (%)	21 (91.3%)	54 (84.4%)	0.41
Prior heart failure, n (%)	15 (65.2%)	34 (53.1%)	0.32
Myocardial infarction, n (%)	7 (30.4%)	20 (31.3%)	0.94
Coronary artery disease, n (%)	12 (52.2%)	27 (42.2%)	0.41
Peripheral artery disease, n (%)	4 (17.4%)	8 (12.5%)	0.56
Tobacco use, n (%)	12 (52.2%)	34 (53.1%)	0.94
Diabetes mellitus, n (%)	9 (39.1%)	33 (51.6%)	0.31
**Concomitant medications**			
Angiotensin II receptor blocker, n (%)	2 (8.7%)	12 (18.8%)	0.26
Angiotensin converting enzyme inhibitor, n (%)	5 (21.7%)	21 (32.8%)	0.32
Beta blocker, n (%)	16 (69.6%)	48 (75.0%)	0.61
Mineralocorticoid receptor antagonist, n (%)	3 (13.0%)	11 (17.2%)	0.64
Thiazide diuretic, n (%)	5 (21.7%)	6 (9.4%)	0.13
Loop diuretic, n (%)	16 (69.6%)	39 (60.9%)	0.46
**Physical Examination**			
Heart rate, beats/min, mean ± SD	84.7 (20.9)	88.5 (26.2)	0.49
Systolic blood pressure, mmHg, mean ± SD	143.1 (32.6)	146.2 (31.2)	0.69
Diastolic blood pressure, mmHg, mean ± SD	74.6 (19.2)	78.0 (16.7)	0.45
Body-mass index, kg/m2, mean ± SD	29.4 (7.2)	31.8 (21.0)	0.59
Jugular venous distension, n (%)	11 (47.8%)	27 (42.2%)	0.64
Hepatojugular reflux, n (%)	1 (4.3%)	11 (17.2%)	0.13
Murmur, n (%)	8 (34.8%)	10 (15.6%)	0.05
Rales on lung exam, n (%)	20 (87.0%)	51 (79.7%)	0.44
Cool extremities, n (%)	3 (5%)	0 (0%)	0.29
S3 gallop, n (%)	0 (0.0%)	4 (6.3%)	0.22
Wheezing, n (%)	6 (26.1%)	7 (10.9%)	0.08
Peripheral edema, n (%)	18 (78.3%)	45 (70.3%)	0.46
**Chest X Ray**			
Interstitial edema, n (%)	13 (56.5%)	35 (54.7%	0.88
Pleural effusion, n (%)	14 (60.9%)	19 (29.7%)	0.008
Infiltrate/pneumonia, n (%)	5 (21.7%)	12 (18.8%)	0.76
**Laboratory testing**			
Creatinine, mg/dL, median [IQR]	1.4 (0.9, 1.8)	1.1 (0.9, 1.6)	0.43
BUN, mg/dL, median [IQR]	33.0 (20.0, 43.0)	25.5 (18.5, 38.0)	0.76
eGFR, mL/min/1.73 m2, median [IQR]	45.6 (29.5, 80.4)	60.3 (36.6, 81.3)	0.25
**ED management**			
Intravenous contrast administration, n (%)	2 (8.7%)	6 (9.4%)	0.92
Loop diuretic drip, n (%)	16 (69.6%)	39 (60.9%)	0.46
Initial intravenous furosemide dose, mg, median [IQR]	20.0 (20.0, 40.0)	40.0 (20.0, 40.0)	0.07

Few differences between the groups existed.

### Biomarkers to predict outcome

**Table 2** details median concentrations of biomarkers in patients as a function of WRF. Though numerical differences were seen, no statistical difference was observed when considering the median values of each biomarker relative to the presence or absence of WRF. We subsequently dichotomized patients with biomarkers above or under the median value of the biomarkers to evaluate for associations in this regard. Once again, patients with or without WRF did not differ for all biomarkers of cardiac stretch, renal function or renal injury.

**Table 2:** Results of baseline biomarkers in patients who developed worsening renal function and those who did not.

	Worsening renal function		
Variable	Yes (N = 26)	No (N = 76)	P
**Biomarkers of cardiac stretch**			
NT-proBNP, pg/mL, median [IQR]	4629 [2320–8620]	3207 [1746–8131]	0.48
BNP, pg/mL, median [IQR]	600 [263–1570]	437 [234–724]	0.20
sST2, ng/mL, median [IQR]	106 [82–182]	101 [69–154]	0.43
**Biomarkers of renal function**			
Screat, mg/L, median [IQR]	1.4 [0.9–1.8]	1.1 [0.8–1.6]	0.21
eGFR, ml/min, median [IQR]	46 [29–80]	60 [37–81]	0.25
**Biomarkers of renal injury**			
NGAL, pg/mL, median [IQR]	233 [149–379]	174 [102–244]	0.13
Urine KIM-1, pg/mL, median [IQR]	963 [182–1547]	681 [208–194]	0.76
Urine NAG, UI/ml, median [IQR]	2.1 [1.1–3.2]	2.4 [1.3–4.1]	0.52
Urine fibrinogen, ng/mL, median [IQR]	46 [14–82]	40 [9–257]	0.76
Urine Cystatin C, ng/mL, median [IQR]	62 [41–114]	97 [34–195]	0.27

Amino-terminal pro-B type natriuretic peptide, NT-proBNP; B type natriuretic peptide, BNP; Estimated glomerular filtration rate, eGFR; Neutrophil gelatinase-associated lipocalin, NGAL, urine kidney injury molecule-1, KIM-1, urine N-acetyl-beta-D-glucosaminidase, NAG. Urine biomarkers are expressed per gram urinary creatinine.

**Figure 1:**
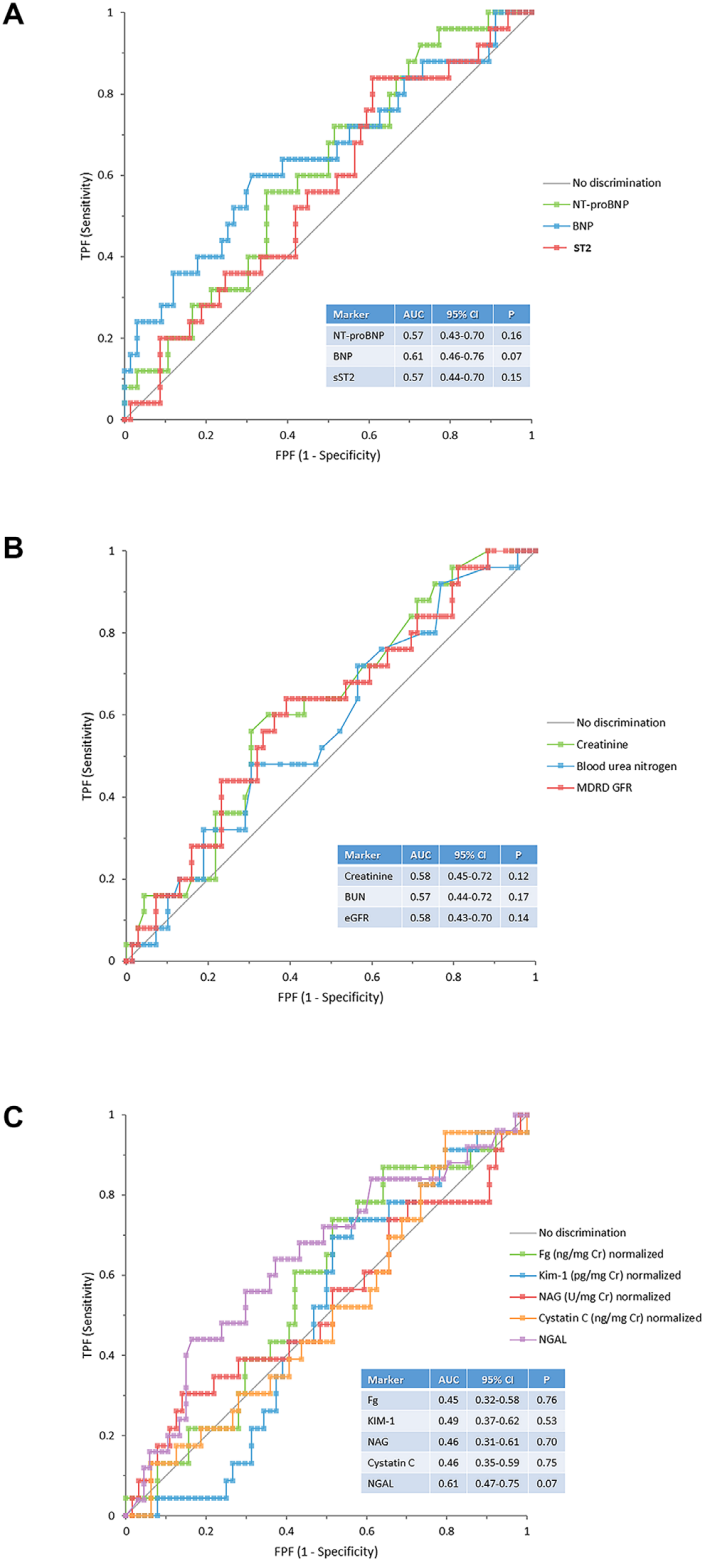
Receiver operating curve of biomarkers of cardiac stretch (1A), renal function (1B) and renal injury (1C).

Results of the ROC curves analysis are presented in Figure 1 and show poor area under the curve for both outcomes examined.

In univariable analyses (Table 3), among biomarkers measured, only BNP was predictive of WRF. In adjusted analyses, no biomarkers remained in the model for predicting outcomes.

**Table 3:** Logistic regression analysis for the primary endpoint of WRF.

Variable	Univariate OR (95% CI) worsening renal function	P-value worsening renal function
**ST2**	1.01 (0.97, 1.06)	0.482
**BNP**	**1.10 (1.01, 1.19)**	**0.022**
**SCreat**	1.35 (0.82, 2.22)	0.246
eGFR	0.90 (0.75, 1.07)	0.229
NAG	1.02 (0.93, 1.11)	0.699
**NGAL**	1.12 (0.93, 1.34)	0.238
NT_proBNP	1.03 (0.99, 1.06)	0.157
Log BNP	1.76 (0.99, 3.13)	0.054
**Log NGAL**	1.60 (0.84, 3.05)	0.153
Log NT-proBNP	1.32 (0.85, 2.06)	0.219
**Log ST2**	1.46 (0.73, 2.91)	0.282
BNP > median	1.89 (0.71, 5.01)	0.201
Cystatin > median	1.38 (0.53, 3.61)	0.507
Urine fibrinogen > median	0.53 (0.20, 1.41)	0.204
Kim-1 > median	1.09 (0.42, 2.83)	0.858
NAG > median	1.16 (0.45, 3.01)	0.759
NGAL > median	1.89 (0.71, 5.01)	0.201
NT-proBNP > median	1.43 (0.55, 3.77)	0.464
ST2 > median	1.88 (0.71, 4.96)	0.204
BNP >553 ng/ml	2.36 (0.89, 6.27)	0.084
**BUN >33 (optimal cut)**	2.02 (0.76, 5.34)	0.158
Creatinine >1.35 mg/l(optimal cut)	2.23 (0.85, 5.89)	0.105
eGFR <60 ml/min (optimal cut)	1.56 (0.59, 4.10)	0.372
NGAL >196 ng/ml (optimal cut)	1.77 (0.67, 4.69)	0.251
NT-proBNP >2840 ng/ml(optimal cut)	1.59 (0.59, 4.30)	0.360

In univariable analysis, several candidates were significant predictors. Optimal cutoffs were determined using the value providing optimal sensitivity and specificity balance. Amino-terminal pro-B type natriuretic peptide, NT-proBNP; B type natriuretic peptide, BNP; Estimated glomerular filtration rate, eGFR; Neutrophil gelatinase-associated lipocalin, NGAL, urine kidney injury molecule-1, KIM-1, urine N-acetyl-beta-D-glucosaminidase, NAG; urine Cystatin C, uCyst C.

### Relationship between renal injury and renal function

Spearman correlation coefficients are presented in Table 4. Interestingly, while plasma NGAL and biomarkers of renal function (BUN and Screat) were correlated, none of the urine biomarkers of renal injury correlated with biomarkers of renal function on admission. Urine biomarkers of renal injury exhibited correlation (but inconsistently) between each other. None of the urine biomarkers of renal injury correlated with plasma NGAL.

**Table 4:** Spearman correlation coefficients between biomarkers.

	NT-proBNP	BNP	Creatinine	BUN	ST2	Blood NGAL	Urinary fibrinogen	Urinary KIM-1	Urinary NAG	Urinary cystatin
**NT-proBNP**	1.00	**0.72 p<0.001**	**0.49 p<0.001**	**0.33 p = 0.002**	**0.40 p<0.001**	**0.35 p = 0.001**	−0.001 p = 0.993	−0.08 p = 0.488	−0.009 p = 0.934	−0.007 p = 0.946
**BNP**	**0.72 p<0.001**	1.00	0.19 p = 0.080	0.07 p = 0.505	0.21 p = 0.055	**0.34 p = 0.001**	−0.14 p = 0.217	−0.16 p = 0.137	−0.06 p = 0.555	0.08 p = 0.484
**Creatinine**	**0.49 p<0.001**	0.19 p = 0.080	1.00	**0.76 p<0.001**	**0.36 p<0.001**	**0.46 p<0.001**	0.20 p = 0.068	0.20 p = 0.064	0.09 p = 0.404	−0.05 p = 0.639
**BUN**	**0.33 p = 0.002**	0.07 p = 0.505	**0.76 p<0.001**	1.00	0.32 p = 0.003	**0.47 p<0.001**	0.11 p = 0.305	0.18 p = 0.093	−0.01 p = 0.900	−0.11 p = 0.318
**ST2**	**0.40 p<0.001**	0.21 p = 0.055	**0.36 p<0.001**	**0.32 p = 0.003**	1.00	**0.37 p<0.001**	0.12 p = 0.259	0.11 p = 0.315	0.11 p = 0.294	0.06 p = 0.584
**Blood NGAL**	**0.35 p = 0.001**	**0.34 p = 0.001**	**0.46 p<0.001**	**0.47 p<0.001**	**0.37 p<0.001**	1.00	−0.02 p = 0.892	−0.13 p = 0.233	0.07 p = 0.547	−0.07 p = 0.544
**Urinary fibrinogen**	−0.001 p = 0.993	−0.14 p = 0.217	0.20 p = 0.068	0.11 p = 0.305	0.12 p = 0.259	−0.02 p = 0.892	1.00	**0.30 p = 0.005**	**0.24 p = 0.023**	**0.34 p = 0.002**
**Urinary KIM-1**	−0.08 p = 0.488	−0.16 p = 0.137	0.20 p = 0.064	0.18 p = 0.093	0.11 p = 0.315	−0.13 p = 0.233	**0.30 p = 0.005**	1.00	0.21 p = 0.053	0.14 p = 0.197
**Urinary NAG**	−0.009 p = 0.934	−0.06 p = 0.555	0.09 p = 0.404	−0.01 p = 0.900	0.11 p = 0.294	0.07 p = 0.547	**0.24 p = 0.023**	0.21 p = 0.053	1.00	0.13 p = 0.248
**Urinary cystatin**	−0.007 p = 0.946	0.08 p = 0.484	−0.05 p = 0.639	−0.11 p = 0.318	0.06 p = 0.584	−0.07 p = 0.544	**0.34 p = 0.002**	0.14 p = 0.197	0.13 p = 0.248	1.00

All values were log transformed except serum creatinine (Screat) and blood urea nitrogen (BUN), NT-proBNP, N-terminal pro-brain natriuretic peptide; BNP, brain natriuretic peptide-BNP; eGFR, estimated glomerular filtration rate; pNGAL, plasma neutrophil gelatinase associated lipocalin; uNGAL, urine neutrophil gelatinase associated lipocalin; KIM-1, kidney injury molecule-1; NAG, urine N-acetyl-beta-D-glucosaminidase.

## Discussion

Altered renal function and WRF are well-established factors associated with poor-prognosis in patients with ADHF. Type 1 CRS, defined as an alteration of renal function in consequence of HF, has therefore been a matter of major interest in trying to better understand its mechanisms and implication in the course of HF patients. CRS emerges from the combination of systemic and intra-renal hemodynamic alterations as well as inflammatory mechanisms [3]. What challenges clinicians monitoring patients with ADHF at risk for CRS is the fact that not all WRF is associated with poor prognosis, and also that the standard tools for recognizing change in renal function (typically serum creatinine or eGFR) lags behind the acute insult to the kidney [10], [11]. Accordingly, the complex mechanisms for CRS, variable phenotypes of WRF, and imperfect tools for its diagnosis make new approaches necessary. Some degree of increase in serum creatinine and hemoconcentration appear to be associated with better outcome compared to patients with no increase in serum creatinine in some observational study. However these observations appear true only with slight elevation in serum creatinine (<20%), only reflecting hemoconcentration but not true decline in GFR. On our cohort, only 3 out of 8 who died had change <+20% in Screat. With the development of biomarkers potentially indicative of renal injury comes the opportunity to potentially better characterize episodes of WRF, thus providing better phenotyping of [12], [13]. In this line it is expected that early recognition of on-going renal injury (i.e. renal damage) may help to anticipate WRF and therefore provide a window for tailored therapeutic interventions.

In this study we included 87 consequent patients admitted in two centers with ADHF and simultaneously measured blood based biomarkers of cardiac function, renal function and renal injury, as well as urine-based biomarkers of renal injury, including urine KIM-1, NAG, cystatin C and fibrinogen. The central finding of our study was that none of the biomarkers of renal function or renal injury was entirely accurate to predict WRF in this population. Surprisingly, patients who subsequently developed WRF did not show worse renal dysfunction or more intense renal injury on admission than patients who did not. Within this small group, the only biomarker that identified risk for WRF was BNP, actually, re-enforcing the role of renal congestion in this setting. Despite the negative result for WRF, we reproduced findings from previous studies showing an association between mortality and renal dysfunction in showing that biomarkers of renal function (i.e. Screat and eGFR) were predictor of WRF.

NGAL is the biomarker of renal injury that has received most attention in critically ill patients as well in patients with HF. NGAL is a member of the lipocalin superfamily of proteins, expressed in various types of cells (including epithelial cells) freely filtered by the glomerulus and thenafter reabsorbed by proximal tubular cells [14]. Plasma NGAL was shown to increase in various conditions including systemic inflammation, cancer or atherosclerosis. Previously studies have yielded conflicting results with regard to predictive accuracy of NGAL in different conditions, including ADHF patients. While some suggested that pNGAL could predict WRF with moderate or good accuracy [15]. Others found that pNGAL [16] was poorly or not predictive of WRF in this setting. In a single center study, Shrestha et al reported that uNGAL had lower AUC than pNGAL for prediction of WRF in ADHF patients [17]. Our results are in the line with results observed in those studies.

While studies of urinary biomarkers have shown evidence of renal injury in patients with chronic HF [18], [19]. Very few studies have focused on urinary biomarkers in ADHF. KIM-1 is a type I transmembrane glycoprotein highly expressed in post-ischemic kidneys and released after acute kidney injury (AKI). Likewise, NAG, a lysosomal brush border enzyme of the proximal tubule cells is released into the urine after renal injury; both may serve as biomarkers of tubular injury, and both may be more specific for renal injury due to venous congestion than NGAL. In a pathophysiologic study, KIM-1 and NAG rapidly increased after diuretic withdrawal (with parallel BNP increase) while NGAL was not affected; both urinary peptides returned to baseline values after reintroduction of diuretic [20]. In a single center study, uNGAL levels were not different on admission between patients with renal dysfunction and those without [21]. Shrestha et al found that uNGAL and pNGAL had similar - but modest - ability to predict WRF (AUC-ROC of 0.64 and 0.67 respectively) [17]. However, in another single center study including 53 patients, Park et al found that uNGAL and KIM-1 levels were not higher in patients with AKI and could not predict recovery from AKI [21]. To the best of our knowledge, our study for the first time explored the predictive value of urine Cystatin C and urine fibrinogen in ADHF patients. Cystatin-C 13.3-kDa non-glycosylated cysteine protease inhibitor produced by all nucleated cells of the body and released at a constant rate, freely filtered by the glomerulus and then reabsorbed by the tubular epithelial cells. Cystatin C detected in the urine is therefore used as a biomarker of tubular dysfunction and injury [22]. Urine fibrinogen has recently been described as a biomarker of ischemic AKI. Fibrinogen is significantly up-regulated after kidney ischemia/reperfusion in rats and humans [23]. However, urine fibrinogen was not predictive of WRF or mortality in our study.

Taken together, consistency of result throughout clinical studies does not appear to support the use of biomarkers of renal injury to predict WRF in patients with ADHF. However, our results add to the evidence that renal injury and renal dysfunction should be view as separate entities in this setting. Interestingly, none of the urine biomarkers of renal injury correlated with biomarkers of renal function, but did show correlations between each other. On the other hand, biomarkers of renal function did correlate with blood-based biomarkers of cardiac stretch (i.e. BNP, NT-proBNP, sST2) and NGAL. Our results suggest patients may have ongoing kidney damage (with constitutive release of biomarkers of renal injury) uncoupled from loss of renal function. These data argue that urine biomarkers provide different type of information that blood biomarkers in ADHF. Urine biomarker could more specifically reflect renal injury than blood biomarkers, but may lack specificity for predicting clinically significant WRF. These observations may arise from the association between rather limited renal injury in this setting and the various renal functional reserves available in those patients. For the same volume of damaged nephrons, loss of renal function would therefore be more pronounced in patients with low or no renal functional reserve compared to patients with intact renal structure and functional reserve. On the opposite, renal function biomarkers were correlated with biomarkers of venous congestion and cardiovascular disease. Altogether, these data strongly supports the contention that WRF in ADHF arises mostly from hemodynamic factors (i.e. venous congestion) and not from profound structural damage to the kidney. Hopefully, these studies will provide additional data to find out whether ADHF-related renal injury should be viewed as an isolated outcome caused by one mechanism or a broader syndrome with various management strategies driven by various clinical or biochemical guides.

Our study suffers from several limitations. First, it was a rather low sample size with possible lack of power to show a statistical association between biomarkers and outcome, especially for mortality. However, increasing the sample size might allow to reach statistical difference, but such a poor predictive value of renal injury biomarkers in this setting are very much unlikely to be relevant to predict WRF from a clinical perceptive (i.e. for the caring physician on an individual basis for his patient). We believe that biomarkers of renal injury should be studied for a different need than to predict WRF in this setting. Still, a large prospective study is needed for a definitive answer on the prognosis value of renal injury biomarkers in ADHF and their use to guide therapeutic interventions [24], [25].

Our study was furthermore prospective and designed with *a priori* assumptions regarding the endpoints. Notably, we studied a mixture of patients with both chronic and de novo HF. Given the potential effects of chronically administered HF therapeutics such as renin-angiotensin-aldosterone inhibitors on risk for WRF, it remains unclear if analysis of more pure cohorts with all of one or the other scenario would provide comparable results. Another issue are the multiple comparisons performed in this relatively small dataset increases the risk for Type I or Type II error. While correction techniques might alleviate this issue, they are not typically used in such datasets of this size. As well, the overall consistent directionality of data reduces the potential for incorrect interpretation. Finally, our study was performed in only two centers that may limit the generalizability of our results.

### Conclusions

In ED patients with ADHF, urine biomarkers of renal injury did not predict WRF. Our data suggest that a weak association exists between renal dysfunction and renal injury in this setting.
